# Consensus Head Acceleration Measurement Practices (CHAMP): Study Design and Statistical Analysis

**DOI:** 10.1007/s10439-022-03101-0

**Published:** 2022-10-17

**Authors:** Steve Rowson, Jason Mihalik, Jillian Urban, Julianne Schmidt, Steve Marshall, Jaroslaw Harezlak, Brian D. Stemper, Mike McCrea, Jim Funk

**Affiliations:** 1 Biomedical Engineering and Mechanics, Virginia Tech, Blacksburg, VA 24060 USA; 2 Exercise and Sport Science, The University of North Carolina, Chapel Hill, NC 27599 USA; 3 Biomedical Engineering, School of Medicine, Wake Forest University, Winston-Salem, NC 27106 USA; 4 Department of Kinesiology, Mary Frances Early College of Education, University of Georgia, Athens, GA 30602 USA; 5 Gillings School of Public Health, The University of North Carolina, Chapel Hill, NC 27599 USA; 6 School of Public Health, Indiana University Bloomington, Bloomington, IN 47405 USA; 7 Joint Department of Biomedical Engineering, Marquette University & Medical College of Wisconsin, Department of Neurosurgery, Medical College of Wisconsin, Neuroscience Research, Zablocki Veterans Affairs Medical Center, Milwaukee, WI 53295 USA; 8 Department of Neurosurgery, Medical College of Wisconsin, Neuroscience Research, Zablocki Veterans Affairs Medical Center, Milwaukee, WI 53295 USA; 9 Biocore LLC, 1627 Quail Run, Charlottesville, VA 22911 USA

**Keywords:** Head impact measurement devices, Head impact sensors, Head acceleration, Sports, Biomechanics, Concussion, Head impact exposure

## Abstract

Head impact measurement devices enable opportunities to collect impact data directly from humans to study topics like concussion biomechanics, head impact exposure and its effects, and concussion risk reduction techniques in sports when paired with other relevant data. With recent advances in head impact measurement devices and cost-effective price points, more and more investigators are using them to study brain health questions. However, as the field's literature grows, the variance in study quality is apparent. This brief paper aims to provide a high-level set of key considerations for the design and analysis of head impact measurement studies that can help avoid flaws introduced by sampling biases, false data, missing data, and confounding factors. We discuss key points through four overarching themes: study design, operational management, data quality, and data analysis.

## Summary Statements

This work was part of the Consensus Head Acceleration Measurement Practices (CHAMP) project. The objective of CHAMP was to develop consensus best practices for gathering, reporting, and analyzing head acceleration measurement data in sports. Subject matter experts were recruited to draft a series of papers on various aspects of the issue. As described in detail in a companion paper,^1^ each team drafted a paper and several summary statements ahead of the CHAMP Conference, held on March 24–25, 2022, at the Children's Hospital of Philadelphia. The purpose of this paper is to provide a high-level set of key considerations for the design and analysis of head impact measurement studies. The following summary statements were discussed, revised as necessary, and ultimately approved by more than 80% of the vote at the conference:

- Head impact sensor studies are typically observational in design, which limits their conclusiveness because they are easily contaminated by unseen confounding factors and biases. Investigators should be wary of selection and sampling biases when composing their samples. Efforts should be made to measure and account for suspected confounders.
- Head impact sensor studies benefit from multidisciplinary teams with essential expertise. In addition, establishing partnerships with the research participant community can help produce more representative and reliable conclusions.
- Proper operational planning for sensor maintenance and technical failures will help minimize missing data. In addition, video recording of data collection sessions is recommended as a resource for explaining and verifying impact events as needed.
- Data quality must be assessed for outliers and spurious data and addressed through data cleaning practices. Suspected missing data should be noted, and all kinematic waveforms should be inspected, either computationally or manually. Sensor validity should dictate the necessity and scope of these practices.
- Investigators should employ analysis techniques that minimize sampling bias effects. Further, we recommend statistical transparency in both procedure and output.
- Investigators should perform a common-sense check on the data and their analysis results. Care should be taken to investigate results that appear to be inconsistent, unrealistic, or counterintuitive. Explanations and disclosures of disparities with reality will help inform better data collection, cleaning, and analysis techniques.

## Introduction

Head impact measurement devices provide an opportunity to collect data directly from humans in real-world impact environments. Studies measuring these impacts can enlighten us on biomechanical mechanisms and tolerances of concussion and teach us about head impact exposure in sporting environments and the ability of interventions to reduce concussion risk. As head impact sensor technology has become more cost-effective, the number of studies being conducted and published has continually grown. Investigators have used head impact sensor data to quantify head impact exposure in sports, relate biomechanical input to clinical outcomes, model biomechanical concussion tolerance, and link repetitive head impact exposure to clinical changes.^2,4,6,7,10–14,16,17,19,28–30,36–38,40–43,45,47^ However, while head impact measurement devices have enabled innovative approaches to answer research questions, study strength and conclusiveness have varied.

This brief paper is not intended to review the existing head impact sensor literature critically. Such reviews exist.^33–35^ Here, we aim to highlight key points for the design and analysis of head impact sensor studies and make recommendations for future studies based on our combined experiences and observations. Our intent is for this paper to serve as a helpful tool in planning and executing head impact studies that are appropriately powered, reproducible, and generalizable. We discuss these points through four areas of concern: study design, operational management, data quality, and data analysis. Broadly considering the topics discussed in each section will help ensure quality results that enable meaningful cross-study analysis and skeptical interpretation of the literature.

## Study Design

A study should be planned from start to finish before any data are collected. However, the temptation to equip a team with sensors and collect data without a predefined research question can be hard to resist. Doing so is likely to produce a dataset that cannot provide meaningful results for even basic objectives like characterizing head impact exposure. This section outlines a high-level approach for designing head impact sensor studies and highlights common pitfalls to avoid.

### Head Impact Sensor Study Design

A head impact sensor study should start with defining the research question(s) to be answered through data collection and analysis. While research questions are often broad, investigators should refine them to a testable hypothesis. There are many brain health questions that head impact measurement devices might have utility in answering. These questions could focus on head impact exposure, concussion biomechanics, injury risk, clinical effects associated with cumulative head impact exposure, risk reduction interventions, and others. Each focus would have its own planned design and considerations to limit bias and confounding effects to maximize study conclusiveness.

With the research question in hand, the investigator must next design the study. There are two classifications of study design: experimental and nonexperimental. Experimental studies involve subjects and conditions that the investigator manipulates. You can think of experimental studies as laboratory experiments where the investigator assigns subjects to conditions. Nonexperimental studies are observational, where the investigator does not assign subjects to conditions but instead observes subjects in naturally occurring conditions. Most head impact sensor studies are nonexperimental given the sound ethical restrictions of human subject research in real-world environments with injury potential. This lack of control creates challenges in interpreting results because confounding factors and biases are often unseen, making observational studies less conclusive.

The investigator should select an appropriate study design to test a study's hypothesis. The investigator should define the variables to be measured and which relationships among them will be studied before the study begins. The variables in head impact sensor studies typically fall into four categories: biomechanical, clinical, conditional, and historical.

1. Biomechanical variables are measured by the sensors and describe the impact event. Linear and rotational skull kinematics are of most interest here, as they are correlated to brain injury mechanisms, and it is not possible to directly measure the brain's impact response in human subjects.^21^ Linear accelerations measured at the accelerometers should be transformed to the head's center of gravity for common reference. The biomechanical variables can either be independent or dependent, depending on the study's question. For example, head acceleration would be a dependent variable in a study comparing head acceleration magnitudes by football player position. Biomechanical exposure variables would be independent variables in a study investigating the correlation between head impact exposure and cognitive test scores.
2. Clinical variables are measured through clinical assessments and describe responses that might be associated with impact. Investigators have studied the effect of head impacts on a wide range of clinical measures, including signs and symptoms, neurocognitive tests, balance, imaging, biomarkers, gait, and oculomotor function.^2,9,14,15,20,25,26,28,46^ Clinical measures are almost always dependent variables that investigators relate to biomechanical measures in head impact sensor studies.
3. Conditional variables are factors that describe the conditions associated with a sample. In the context of head impact measurements in soccer players, examples of conditional variables would include whether an impact was collected in a game or practice, player position, and if the head impact was associated with a header or collision. Conditional variables are included in statistical models to explain some of the variance in the dependent variable. They can be categorical, as described above, or continuous (such as game temperature or playing time) and are modeled as independent variables.
4. Historical variables describe information about the individual. These can include anthropomorphic measurements (such as height and weight), demographic information (such as age and education), and health history (such as learning disorders and concussion history). Like conditional variables, historical variables are factors included in models to explain some of the variance in the dependent variable. They can be categorical or continuous and are modeled as independent variables.

Next, the investigator should consider the nature of planned comparisons. For example, can the hypothesis be tested by comparing two samples? If so, are the samples paired? Is there a multisample hypothesis? How many factors will be considered, and how will multiple comparisons be performed? Does the study have a repeated measures design? Answers to such questions will help identify the most appropriate statistical model. Paired and repeated measures approaches can help increase power and reduce required sample sizes. Examples of paired study designs include matching concussed subjects to control subjects to compare head impact exposure.^36,42^ Examples of repeated measures study designs include clinical assessments performed at multiple time points and compared to head impact exposure measures.^27^

### Sampling

The investigator must determine how the sample will be composed. The most obvious question is: what is an appropriate sample size for the question at hand? The answer is not obvious and depends on the research question and study design. One key consideration is whether the study's research question focuses on head impact exposure or concussion. Sample size in head impact sensor studies typically refers to the number of subjects instrumented or the number of concussions observed. If the study focuses on exposure, equipping 100 subjects with head impact measurement devices might provide 100 useful samples. We state that 100 samples might be produced, even though hundreds of impacts might be collected for each player, because data analyses should reduce each player’s impact data to summary statistics when pooling data between subjects. If the study focuses on concussions, equipping 100 subjects with head impact measurement devices might only yield 5 useful samples over a season (subjects that sustained concussions and completed all testing). The investigator should perform a power analysis using the chosen statistical model and available data from the literature to estimate the sample size required to answer the research question. If injury is being studied, the investigator must incorporate the expected concussion rate into their power analysis. Simulation is useful for complex statistical models without clear power calculations for estimating sample size. We recommend using effect sizes you suspect to have clinical meaning in any power analyses. For instance, detecting a 1 g difference in head impact exposure between position groups within a sport is likely not a meaningful difference.

Another question is: who will make up the sample? In practice, an investigator focuses on a sport they want to measure head impacts in and finds a team to participate. These are typically convenience samples as it is logistically impossible to randomly sample subjects from random teams across the population of interest. The sample is never random in observational head impact sensor studies, which affects generalizability by introducing selection bias. The samples chosen can be too specialized. For example, results from a study on National Football League players cannot be generalized and applied to high school football players. In addition, a study quantifying head impact exposure in soccer players that only enrolled a men's soccer teams could not be generalized to women's soccer. One must consider factors like the sex, level of play, age, and region of enrolled teams when assessing selection bias and the generalizability of their conclusions.

An additional concern is sampling bias. Sampling bias can exist in different forms but pull results away from representative averages. Once a team (or set of teams) are recruited, the investigator enrolls subjects into the study. The investigator might target specific subjects to enroll for various reasons like playing time and player position. On the other hand, subjects with certain characteristics might be more or less likely to agree to participate. These two scenarios might overrepresent one subject subtype and underrepresent another. An example for a study on football player head impact exposure would be where a disproportionate number of skill position players were instrumented relative to lineman. As a result, data analysis results wouldn't represent the team and would be skewed toward skill positions. Uniform sampling of participants and collecting data relevant to the research question are essential aspects of study design that minimize imbalances that mask the truth. However, sampling bias will almost always be present, so data analysis techniques that minimize bias effects should be employed.

### Confounding Factors

Investigators must carefully consider factors that might confound their results. Confounding factors are variables related to the variables of interest that can lead to false conclusions. A confounding variable must be associated with both the main independent variable of interest and the outcome of interest without being on the causal pathway between the two variables. An example would be football player position while investigating the relationship between body mass (independent variable) and linear head acceleration (dependent variable). Player position would act as a confounder because, for example, wide receivers have lower body mass than linemen and the nature and frequency of contact differs between wide receivers and linemen. Conditional and historical variables often describe confounding factors. Conditional variables like player position, depth chart, and session type can influence biomechanical measurements.^12,40,43^ Historical variables like neurologic disorders and concussion history can influence clinical measurements and injury response. Further complicating this, factors like concussion history, head impact exposure history, genetics, resilience, sex, and fitness level likely influence concussion risk and severity in ways that are yet to be defined and are, in some cases, difficult to quantify during baseline assessments. Investigators should consider what factors might influence their variables and make efforts to measure them. Confounding factors can be used as exclusion criteria or be built into statistical models as covariates.

### Instruments

The final step of study design is to select the instruments used to collect data. Various instruments are typically used within a single study. Biomechanical data are captured with head impact measurement devices. Clinical data are captured through a broad range of clinical assessments depending on the study's question. Surveys are often used to capture conditional and historical data. We will focus on head impact measurement devices here, but it is important to note that all instruments have some degree of error. This error presents itself through a combination of systematic and random errors. Systematic error is extremely problematic because there is no simple process to account for it. Systematic error is one-directional, meaning that measurements tend to be consistently higher or lower than truth. For example, a head impact sensor might always overestimate head acceleration and overcount head impact events due to poor coupling with the skull. Random error is less problematic, assuming that its magnitude is not unreasonably high. Random error produces less precise data. It is as likely to be in one direction as the other, allowing error to be averaged out over many measurements. Given certain assumptions about the error distribution, some statistical approaches account for data with random error. With that stated, high random error is problematic for interpreting individual measurements. The investigator should understand the error characteristics of any measurement devices that will be employed. Various works have previously quantified error for existing head impact measurement devices,^5,22,31,32,39,44^ and CHAMP has two papers dedicated to head impact sensor error assessments that we point to here for brevity.^18,24^

## Operational Management

Outside of study design, proper operational management is critical for collecting quality data to support research questions. Here, we identify a few study execution considerations derived from our experiences to help maximize the chances of completing a successful study.

### Investigator Team

Head impact studies are large endeavors that benefit from multidisciplinary teams comprised of biomechanical, clinical, and statistical expertise. A team with relevant knowledge and experience is more likely to collect valid data for each instrument, identify problems, perform sound analysis, and provide thoughtful interpretation than a team without expertise in an essential area. In multi-site studies, uniform biomechanical and clinical data collection methods are needed to pool data. Further, common diagnostic criteria between clinical team members must be agreed upon and implemented. Research teams would also benefit from establishing partnerships with the research participant community. Such partnerships help investigators understand the participant's perspective and conduct more representative and reliable studies. Furthermore, they are the ones on the ground that can help identify the most feasible way to carry out the research study.

### Missing Data

Minimizing missing data should be a priority for investigators. Challenges with participant buy-in and compliance for properly wearing head impact measurement devices can result in missing or low-quality data. Beyond compliance, instrument reliability varies. Collecting consistent day-to-day data is difficult due to device failures, battery charging issues, and participants fiddling with or removing devices. All potential problems create opportunities for missing data. Given that head impact sensor studies tend to require large sample sizes, investigators should ensure that the research team has enough personnel to maintain devices, monitor the system during use, observe and record sessions, and inspect data daily. Inevitable problems can be identified quickly and resolved, minimizing the impact of missing data.

### Video Recording

Recording video of all sessions is another best practice that we encourage. Ideally, video of all players at all times would be recorded using multiple cameras. The video should be deliberately time-synchronized with the sensor equipment. It is often helpful to capture the game clock and scoreboard with video, as well. Video can be used to identify measurements not associated with impact (false-positive measurements), identify impacts not measured by the sensor (false-negative measurements), and provide a means of classifying impact characteristics.

## Data Quality

With data collection complete, data quality must be assessed and maximized before data analysis. The first step is data cleaning. Data cleaning includes filtering out non-impact-related events measured by devices, filtering out events that are not relevant to the research question, identification and removal of outliers, identification and removal of spurious kinematic signatures, and identification and notation of missing data. We recommend that these processes be done regularly throughout data collection to check for systematic issues and allow any problems to be identified and corrected early in the study. Many of these processes rely on instrument validity, which should be quantified from in-lab and on-field evaluations and available to investigators. Some sensor systems employ proprietary software algorithms to clean data, but these vary in their ability to do so correctly. It is up to the investigator to determine which data cleaning steps are necessary for their chosen instrument.

### Video Verification

Video review can be a valuable tool in identifying recordings not related to head impact. Non-impact recordings, such as those associated with participants removing measurement devices or seemingly random event triggering, should be removed from the dataset if they are present. Using video to note sessions' start, end, and break times can provide a simple first filter to remove measurements not associated with play. Then, the remaining events recorded can be cross-referenced with video to identify if they are associated with impact events. The investigator should decide the necessity and intensity of video review based on their study's purpose and instrument quality as determined in validation studies. Again, we point to the CHAMP validation papers on this topic for thorough treatment.^18,24^

### Impact Classification

Investigators should define what they consider a relevant acceleration event for the research question. Sensor systems typically apply a threshold filter to remove low-magnitude events associated with dynamic movements. While there can be a good reason to vary the threshold between studies, differing thresholds make head impact study comparisons difficult. For example, lowering a recording threshold from 20 to 10 g in college football might double the number of impacts recorded for each player. Furthermore, the research question should dictate if all impact-related events are being investigated or if just head impacts are. The investigator should consider removing any measurements not associated with direct head impact if the latter. Video analysis can be used to determine if head accelerations events are associated with body collisions or direct head impacts.

### Identifying Missing Data

Sometimes an instrument will fail to record kinematic data for a head impact. Such events are often noticed when observing an injury or interesting impact and finding no corresponding instrumentation data. In the context of data recording, these are classified as false-negative measurements. Tracking false-negative impact recordings can be an important step in data quality assessment, depending on the research question. Unfortunately, identifying all false-negative measurements is extremely challenging. Tedious video review is required to observe all of the head impact events experienced by the instrumented athletes over the periods of interest. These head impacts then need to be cross-referenced with instrumentation data to identify false negatives. Even then, they are only suspected false negatives because it is not known whether the head impact observed on video was severe enough to trigger the recording of sensor data in the first place. Although tedious, a video review of all head impacts is required to know the false-negative rate of a wearable sensor system. This process need not be performed in every study. Often, only injurious head impacts are verified with video. However, if a full video review of all head impacts is not performed, the authors should reference a validation study in which such a video review was performed. If the authors are using a sensor system that has never been validated with video review, they must acknowledge that the false-negative rate of their sensor system is unknown. Likewise, it is important to acknowledge that most datasets will contain missing data in this form, and the investigator should consider the effects of missing data on their analysis.

### Waveform Review

Beyond video review, kinematic waveform review can be performed to identify and remove erroneous data. The focus here is on kinematic time history and magnitude error. Low-quality waveforms can indicate a coupling problem between the instrument and participant.^48^ Like video review, there a multiple levels of waveform review. The first action would be to identify measurements with unusually high magnitudes. “Unusually high” might be defined as anything over 100 g but should vary depending on the loading environment. While acceleration magnitudes this high occur in sports, they represent a small fraction of the data collected and are likely to be associated with injury if confirmed. Identified outliers can be compared to video and flagged or removed if they are in stark contrast to the observed impact severity. Directionality of the acceleration vector and observed impact direction of force can also be compared to assess waveform validity. Finally, waveforms should be reviewed to identify spurious impacts—those with time-clipped impulses, ringing, multiple nonsensical impulses, and poor signal-to-noise ratios.^23^ Fortunately, these processes can be automated to clean data or flag a small sample of impacts for manual inspection.

## Data Analysis

There is a need for the transparent and consistent reporting of data. Studies tend to use different technologies, compute quantities differently, and apply different statistical methods, all of which make interpreting and comparing studies challenging. This section will highlight a few key sources of variability and suggest best practices where appropriate.

### Biomechanical Variables

Newer instruments are adept at measuring rotational head kinematics, using angular accelerometers, angular rate sensors, or linear accelerometers. Angular acceleration is an absolute measurement that can be characterized by its peak magnitude; however, the head's peak rotational velocity might be less biomechanically meaningful than the change in rotational velocity associated with impact.^3^ Many head impact sensor systems report peak rotational velocity by default, and investigators should take care to correct this variable to the change in velocity associated with impact.

Many head impact studies investigate the effects of repetitive head impact exposure. A simple definition of repetitive head impact exposure is the combined biomechanical load of all head impacts experienced over a given period. The combined biomechanical load is a function of impact frequency and kinematic magnitude. Studies have attempted to compute cumulative exposure in various ways, including the number of impacts, number of impacts paired with 95th percentile acceleration magnitudes, summed accelerations, head impact density measures, and risk-weighted exposure.^8,13,14,36,42^ However, there is no consensus on the optimal method to summarize the cumulative biomechanical load of many head impacts.

### Summarizing Data

A single study might measure hundreds of thousands of head impacts. How an investigator summarizes these data depends on the research question. Here, we note some data characteristics and practices to handle head impact data. It makes sense to perform analyses on a per-player basis in many instances. That means that data are summarized for each subject before cohort values are computed. There are a couple of benefits in doing so. First, it allows variation between participants to be quantified. Second, it mitigates a form of sampling bias where subjects with more head impacts influence the cohort summary values more than subjects with few impacts. At the subject level, distributions of acceleration magnitudes will likely be right-skewed. For heavily skewed distributions, medians and interquartile ranges are often better central tendency and variance measures than averages and standard deviations. 95th percentiles have frequently been used to describe higher magnitude accelerations for participants, assuming each subject has experienced enough impacts. Distributions of summary statistics between subjects should approach normality given a large enough sample and the absence of bias. Each study's approach to summarizing data will vary depending on the research question but should consider the underlying distributions and opportunities to identify and account for bias.

### Statistical Reporting

Investigators should specify their statistical approach during study design. More transparency in statistical methods and output reporting in the literature would be helpful. For example, only reporting p-values is not helpful to the reader for interpreting results. A *p* value is a composite number that combines effect size and precision, yet describes neither. In addition to *p* values, reporting effect size and a confidence interval would better describe a study's statistical findings. Such an approach could prevent small effect sizes with little clinical meaning being overstated due to high precision. The opposite can be a problem too, where large effects might be completely ignored due to low precision. The presence or absence of statistical significance does not reflect impairment (or some other clinical characteristic). It is up to the investigators to interpret any effects and their clinical implications. The appropriate parameter to measure effect size should be identified before the study starts to avoid the temptation to "p-hack" after the fact and scour the data for other parameters that show significant (but unexpected and potentially spurious) effects. Furthermore, it is imperative that investigators use and report appropriate statistical procedures that account for history and regression effects in studies with repeated measure designs. Not doing so threatens a study’s internal validity, which is the confidence level in a cause-and-effect relationship. Such designs typically have subjects complete clinical tests at multiple timepoints.

### Sampling Bias

Investigators should consider what sampling biases might be in their data. If sampling can be shown to minimize biases, estimates can be computed on an absolute basis. If biases exist, methods to deal with them should be implemented, like normalization or reweighting to prevent a subset of subjects or conditions from pulling the data away from representative averages. Most datasets will have some sampling bias in them. One example would come from nonuniform sampling of player position.

Differential subject loss also affects study validity by introducing sampling bias. Head impact sensor studies occur over time, and subjects might drop out of the study before its conclusion. This can be problematic because the attrition might not be random and could differ between the groups being compared. For example, subjects might drop out of a study because they sustained a concussion. This is a form of sampling bias that can develop throughout a study. The method used to account for bias needs to be determined in the context of the research question.

### Dealing with Missing Data

Missing data can be pervasive in head impact sensor datasets. Missing data are almost guaranteed to be in all head impact sensor datasets, whether from device failures, player compliance and participation, or data cleaning. Imputation and removal are two primary methods of dealing with missing data. Imputation involves making an educated guess and filling in missing data. Imputation approaches can make sense in scenarios where one might aim to quantify total head impact exposure over a season but had several sessions where the devices were not functioning correctly. Removal involves removing sample units with incomplete data from the analysis and only analyzing sample units with complete data. Investigators should employ a method compatible with their research question.

### Gut Check

Our last note is to have investigators perform a common-sense check before submitting results for publication. If the results are nonsensical or disagree with what one would expect, care should be taken to confirm the findings. Examples include unrealistically high impact counts for low contact sports and overly high kinematic magnitudes compared to observation. Publication of nonsensical results can largely be prevented through proper study design and data cleaning practices.

## References

[1] Arbogast KB, Caccese JB, Buckley TA, McIntosh A, Henderson K, Stemper BD, Solomon GS, Broglio SP, Funk JR (2022). Consensus head acceleration measurement practices (champ): origins, methods, transparency and disclosure. Ann. Biomed. Eng..

[2] Bahrami N, Sharma D, Rosenthal S, Davenport EM, Urban JE, Wagner B, Jung Y, Vaughan CG, Gioia GA, Stitzel JD (2016). Subconcussive head impact exposure and white matter tract changes over a single season of youth football. Radiology..

[3] Bailey AM, Sherwood CP, Funk JR, Crandall JR, Carter N, Hessel D, Beier S, Neale W (2020). Characterization of concussive events in professional american football using videogrammetry. Ann. Biomed. Eng..

[4] Bartsch AJ, Hedin D, Alberts J, Benzel EC, Cruickshank J, Gray RS, Cameron K, Houston MN, Rooks T, McGinty G (2020). High energy side and rear american football head impacts cause obvious performance decrement on video. Ann. Biomed. Eng..

[5] Beckwith JG, Greenwald RM, Chu JJ (2012). Measuring head kinematics in football: correlation between the head impact telemetry system and hybrid iii headform. Ann. Biomed. Eng..

[6] Beckwith JG, Greenwald RM, Chu JJ, Crisco JJ, Rowson S, Duma SM, Broglio SP, McAllister TW, Guskiewicz KM, Mihalik JP, Anderson S, Schnebel B, Brolinson PG, Collins MW (2013). Head impact exposure sustained by football players on days of diagnosed concussion. Med. Sci. Sports Exerc..

[7] Beckwith JG, Greenwald RM, Chu JJ, Crisco JJ, Rowson S, Duma SM, Broglio SP, McAllister TW, Guskiewicz KM, Mihalik JP, Anderson S, Schnebel B, Brolinson PG, Collins MW (2013). Timing of concussion diagnosis is related to head impact exposure prior to injury. Med Sci Sports Exerc..

[8] Broglio SP, Lapointe A, O'Connor KL, McCrea M (2017). Head impact density: a model to explain the elusive concussion threshold. J. Neurotrauma.

[9] Caccese JB, Best C, Lamond LC, DiFabio M, Kaminski TW, Watson D, Getchell N, Buckley TA (2019). Effects of repetitive head impacts on a concussion assessment battery. Med. Sci. Sports Exerc..

[10] Camarillo DB, Shull PB, Mattson J, Shultz R, Garza D (2013). An instrumented mouthguard for measuring linear and angular head impact kinematics in american football. Ann. Biomed. Eng..

[11] Campolettano ET, Gellner RA, Smith EP, Bellamkonda S, Tierney CT, Crisco JJ, Jones DA, Kelley ME, Urban JE, Stitzel JD, Genemaras A, Beckwith JG, Greenwald RM, Maerlender AC, Brolinson PG, Duma SM, Rowson S (2020). Development of a concussion risk function for a youth population using head linear and rotational acceleration. Ann. Biomed. Eng..

[12] Campolettano ET, Rowson S, Duma SM, Stemper BD, Shah A, Harezlak J, Riggen LD, Mihalik JP, Brooks A, Cameron KL, Giza CC, McAllister TA, Broglio SP, McCrea M (2019). Factors affecting head impact exposure in college football practices: a multi-institutional study. Ann. Biomed. Eng..

[13] Crisco JJ, Wilcox BJ, Beckwith JG, Chu JJ, Duhaime AC, Rowson S, Duma SM, Maerlender AC, McAllister TW, Greenwald RM (2011). Head impact exposure in collegiate football players. J. Biomech..

[14] Davenport EM, Whitlow CT, Urban JE, Espeland MA, Jung Y, Rosenbaum DA, Gioia GA, Powers AK, Stitzel JD, Maldjian JA (2014). Abnormal white matter integrity related to head impact exposure in a season of high school varsity football. J. Neurotrauma.

[15] Duhaime AC, Beckwith JG, Maerlender AC, McAllister TW, Crisco JJ, Duma SM, Brolinson PG, Rowson S, Flashman LA, Chu JJ, Greenwald RM (2012). Spectrum of acute clinical characteristics of diagnosed concussions in college athletes wearing instrumented helmets. J Neurosurg..

[16] Duma SM, Manoogian SJ, Bussone WR, Brolinson PG, Goforth MW, Donnenwerth JJ, Greenwald RM, Chu JJ, Crisco JJ (2005). Analysis of real-time head accelerations in collegiate football players. Clin. J. Sport Med..

[17] Eckner JT, O’Connor KL, Broglio SP, Ashton-Miller JA (2018). Comparison of head impact exposure between male and female high school ice hockey athletes. Am. J. Sports Med..

[18] Gabler L, Patton D, Begonia M, Daniel R, Rezaei A, Huber C, Siegmund G, Rooks T, Wu L (2022). Consensus head acceleration measurement practices (champ): laboratory validation of wearable head kinematic devices. Ann. Biomed. Eng..

[19] Greenwald RM, Gwin JT, Chu JJ, Crisco JJ (2008). Head impact severity measures for evaluating mild traumatic brain injury risk exposure. Neurosurgery.

[20] Gysland SM, Mihalik JP, Register-Mihalik JK, Trulock SC, Shields EW, Guskiewicz KM (2012). The relationship between subconcussive impacts and concussion history on clinical measures of neurologic function in collegiate football players. Ann. Biomed. Eng..

[21] Hardy WN, Mason MJ, Foster CD, Shah CS, Kopacz JM, Yang KH, King AI, Bishop J, Bey M, Anderst W, Tashman S (2007). A study of the response of the human cadaver head to impact. Stapp Car Crash J..

[22] Kieffer EE, Begonia MT, Tyson AM, Rowson S (2020). A two-phased approach to quantifying head impact sensor accuracy: in-laboratory and on-field assessments. Ann. Biomed. Eng..

[23] Kieffer EE, Vaillancourt C, Brolinson PG, Rowson S (2020). Using in-mouth sensors to measure head kinematics in rugby. IRCOBI Conf..

[24] Kuo C, Patton D, Rooks T, Tierney G, McIntosh A, Lynall R, Esquivel A, Daniel R, Kaminski T, Mihalik J (2022). On-field deployment and validation for wearable devices. Ann. Biomed. Eng..

[25] Maerlender AC, Brolinson PG, Crisco JJ, Urban J, Ajamil A, Rowson S, Campolettano ET, Gellner RA, Bellamkonda S, Kieffer E (2020). Neuropsychological change after a single season of head impact exposure in youth football. J. Int. Neuropsychol. Soc..

[26] McAllister TW, Flashman LA, Maerlender A, Greenwald RM, Beckwith JG, Tosteson TD, Crisco JJ, Brolinson PG, Duma SM, Duhaime AC, Grove MR, Turco JH (2012). Cognitive effects of one season of head impacts in a cohort of collegiate contact sport athletes. Neurology.

[27] McCaffrey MA, Mihalik JP, Crowell DH, Shields EW, Guskiewicz KM (2007). Measurement of head impacts in collegiate football players: clinical measures of concussion after high- and low-magnitude impacts. Neurosurgery.

[28] McCrea M, Broglio SP, McAllister TW, Gill J, Giza CC, Huber DL, Harezlak J, Cameron KL, Houston MN, McGinty G, Jackson JC, Guskiewicz K, Mihalik J, Brooks MA, Duma S, Rowson S, Nelson LD, Pasquina P, Meier TB, a. t. C. C. Investigators (2020). Association of blood biomarkers with acute sport-related concussion in collegiate athletes: findings from the NCAA and department of defense care consortium. JAMA Network Open.

[29] McCrea MA, Shah A, Duma S, Rowson S, Harezlak J, McAllister TW, Broglio SP, Giza CC, Goldman J, Cameron KL, Houston MN, McGinty G, Jackson J, Guskiewicz K, Mihalik JP, Brooks A, Pasquina P, Stemper BD (2021). Opportunities for prevention of concussion and repetitive head impact exposure in college football players: a concussion assessment, research, and education (care) consortium study. JAMA Neurol..

[30] Mihalik JP, Chandran A, Powell JR, Roby PR, Guskiewicz KM, Stemper BD, Shah AS, Rowson S, Duma S, Harezlak J (2020). Do head injury biomechanics predict concussion clinical recovery in college american football players?. Ann. Biomed. Eng..

[31] Nevins D, Hildenbrand K, Kensrud J, Vasavada A, Smith L (2016). Field evaluation of a small form-factor head impact sensor for use in soccer. Procedia Eng..

[32] Nevins D, Smith L, Kensrud J (2015). Laboratory evaluation of wireless head impact sensor. Procedia Eng..

[33] O'Connor KL, Rowson S, Duma SM, Broglio SP (2017). Head-impact–measurement devices: a systematic review. J. Athl. Train..

[34] Patton DA, Huber CM, Jain D, Myers RK, McDonald CC, Margulies SS, Master CL, Arbogast KB (2020). Head impact sensor studies in sports: a systematic review of exposure confirmation methods. Ann. Biomed. Eng..

[35] Rowson B, Duma SM (2020). A review of on-field investigations into the biomechanics of concussion in football and translation to head injury mitigation strategies. Ann. Biomed. Eng..

[36] Rowson S, Campolettano ET, Duma SM, Stemper B, Shah A, Harezlak J, Riggen L, Mihalik JP, Guskiewicz KM, Giza C (2019). Accounting for variance in concussion tolerance between individuals: comparing head accelerations between concussed and physically matched control subjects. Ann. Biomed. Eng..

[37] Rowson S, Duma SM, Beckwith JG, Chu JJ, Greenwald RM, Crisco JJ, Brolinson PG, Duhaime AC, McAllister TW, Maerlender AC (2012). Rotational head kinematics in football impacts: an injury risk function for concussion. Ann. Biomed. Eng..

[38] Rowson S, Duma SM, Stemper BD, Shah A, Mihalik JP, Harezlak J, Riggen LD, Giza CC, DiFiori JP, Brooks A (2018). Correlation of concussion symptom profile with head impact biomechanics: a case for individual-specific injury tolerance. J. Neurotrauma.

[39] Siegmund GP, Guskiewicz KM, Marshall SW, DeMarco AL, Bonin SJ (2016). Laboratory validation of two wearable sensor systems for measuring head impact severity in football players. Ann. Biomed. Eng..

[40] Stemper BD, Harezlak J, Shah AS, Rowson S, Mihalik JP, Riggen L, Duma S, Pasquina P, Broglio SP, McAllister TW (2022). Association between preseason/regular season head impact exposure and concussion incidence in ncaa football. Med. Sci. Sports Exerc..

[41] Stemper BD, Shah AS, Harezlak J, Rowson S, Duma S, Mihalik JP, Riggen LD, Brooks A, Cameron KL, Giza CC (2019). Repetitive head impact exposure in college football following an ncaa rule change to eliminate two-a-day preseason practices: a study from the ncaa-dod care consortium. Ann. Biomed. Eng..

[42] Stemper BD, Shah AS, Harezlak J, Rowson S, Mihalik JP, Duma SM, Riggen LD, Brooks A, Cameron KL, Campbell D (2018). Comparison of head impact exposure between concussed football athletes and matched controls: evidence for a possible second mechanism of sport-related concussion. Ann. Biomed. Eng..

[43] Stemper BD, Shah AS, Mihalik JP, Harezlak J, Rowson S, Duma S, Riggen LD, Brooks A, Cameron KL, Giza CC (2020). Head impact exposure in college football following a reduction in preseason practices. Med. Sci. Sports Exerc..

[44] Tyson AM, Duma SM, Rowson S (2018). Laboratory evaluation of low-cost wearable sensors for measuring head impacts in sports. J. Appl. Biomech..

[45] Urban JE, Davenport EM, Golman AJ, Maldjian JA, Whitlow CT, Powers AK, Stitzel JD (2013). Head impact exposure in youth football: high school ages 14 to 18 years and cumulative impact analysis. Ann. Biomed. Eng..

[46] Vernau BT, Grady MF, Goodman A, Wiebe DJ, Basta L, Park Y, Arbogast KB, Master CL (2015). Oculomotor and neurocognitive assessment of youth ice hockey players: baseline associations and observations after concussion. Dev. Neuropsychol..

[47] Wilcox BJ, Beckwith JG, Greenwald RM, Chu JJ, McAllister TW, Flashman LA, Maerlender AC, Duhaime A-C, Crisco JJ (2014). Head impact exposure in male and female collegiate ice hockey players. J. Biomech..

[48] Wu LC, Nangia V, Bui K, Hammoor B, Kurt M, Hernandez F, Kuo C, Camarillo DB (2016). In vivo evaluation of wearable head impact sensors. Ann. Biomed. Eng..

